# Bills of Mortality and the Birth of Epidemic Surveillance: Evidence, Uncertainty, and Decision-Making in Defoe’s Plague Narrative

**DOI:** 10.7759/cureus.108532

**Published:** 2026-05-09

**Authors:** Eleni Tatsiou, Marianna Karamanou

**Affiliations:** 1 Department of History of Medicine and Medical Ethics, School of Medicine, National and Kapodistrian University of Athens, Athens, GRC

**Keywords:** black plague, covid-19, defoe, medicine history, public health

## Abstract

By drawing parallels rather than following events exactly, we interpret Daniel Defoe's most important work as a literary response to the Bills of Mortality that foreshadows enduring concerns of epidemic surveillance. These include the aggregation of deaths in time and space, the interpretation of trends, and a persistent awareness of bias and uncertainty. We examine how Defoe’s narrator "Henry Foe (H.F.)" reads, quotes, compares, and questions the weekly bills in the context of the Great Plague of London in 1665. He treats numerical data neither as transparent reality nor as mere ornament, but as contested evidence shaped by under-registration, diagnostic ambiguity, and pressure to minimize public alarm. Through close reading of selected passages, we show how H.F. juxtaposes printed figures with street-level observation, theological and humoral explanations, and contemporary debates on miasma, contagion, and quarantine. We do not claim that Defoe practiced surveillance in any modern sense. Rather, we argue that his narrative illuminates recurrent ethical and epistemological questions about data quality, transparency, trust, and the communication of risk. These questions have become newly salient amid recent preoccupations with dashboards, case counts, and excess mortality during the COVID-19 pandemic. Defoe's journal thus offers historians of medicine and public health a sophisticated early case study of the promises and pitfalls of mortality statistics.

## Editorial

Introduction

A Journal of the Plague Year by Daniel Defoe (1722) is among the most enduring literary representations of the Great Plague of London (1665) [1]. For three centuries, it has occupied an ambiguous position between eyewitness chronicle, historical reconstruction, and imaginative fiction [2,3]. The text offers a detailed account of how the plague reshaped the "face" of London, its streets, parishes, and households, while the narrator known only as "Henry Foe (H.F.)" interrogates the causes, responsibilities, and appropriate measures taken against the disease [1,4]. One of the work's most striking features, repeatedly noted by editors and historians, is Defoe's systematic use of numerical data, such as tables of deaths, weekly catalogs of burials, and sustained reference to the Bills of Mortality as instruments for tracking the epidemic's progress.

Defoe was not writing simply about 1665. Composed 57 years after the events it describes, the journal also reflects the anxieties of the 1720s, particularly the threat of plague arriving from Marseilles and the contemporary debates over quarantine and the shutting up of houses [1-3]. Defoe drew on printed Bills of Mortality, municipal orders, medical treatises, and oral testimony, weaving them into a narrative intended both to instruct and to warn future readers [1,3,5]. Numerical evidence sits at the center of his representation. H.F. follows the weekly Bills as they rise and fall, compares parishes, comments on suspected under-reporting, and reflects on how ordinary Londoners and civic authorities read these numbers as signals of risk, safety, or impending catastrophe [1,6,7].

Seventeenth-century London had developed increasingly sophisticated mechanisms for monitoring mortality [2,8,9]. The Bills of Mortality were compiled weekly, tabulating deaths by cause and parish. Printed, posted in public places, and sold to subscribers, they served a dual function. They informed individual decisions about whether to flee, stay, or alter daily movements, and they supported civic deliberations on quarantine, house closure, and the provision of relief [4,8]. From the perspective of the history of epidemiology, the Bills can be read as an early, imperfect, but influential method for transforming scattered deaths into a legible pattern of epidemic spread [10,11]. Historians have nevertheless repeatedly underscored their limitations and biases, including the exclusion of certain religious groups and of the urban poor, the concealment of plague deaths under other diagnostic labels, and the exertion of political pressure to minimize alarm [8].

Within this context, A Journal of the Plague Year is an invaluable source for examining how an early 18th-century observer conceptualized epidemic numbers. Defoe's narrator does not treat the Bills of Mortality as transparent mirrors of reality. He interrogates their completeness, speculates about hidden mortality, and juxtaposes the printed figures with his own observations as he walks the streets and parishes of London [1]. His narrative thus anticipates, again by analogy rather than direct lineage, several features of later epidemic surveillance. These include the aggregation of deaths across time and space, the interpretation of trends as evidence for the presence or absence of contagion, and a persistent awareness of uncertainty, under-reporting, and misclassification [3,5]. At the same time, Defoe's reflections remain embedded within contemporary medical and moral frameworks. H.F. engages with debates about miasma and contagion, invisible "effluvia" and "worms," and divine judgment and repentance. Numerical evidence never stands alone in the Journal. It is constantly interpreted through theological, humoral, and experiential lenses [1,4].

This editorial adopts an interpretive, close-reading approach rather than a systematic textual analysis. We selected passages from A Journal of the Plague Year in which H.F. explicitly engages with numerical data from the Bills of Mortality, including moments where he cites specific figures, questions their accuracy, performs ad hoc corrections for suspected under-reporting, or connects the printed numbers to his own observations in the streets. Quotations are drawn from the 1722 first edition as reproduced in the Brayley 1876 revision. These passages are placed in dialogue with recent historiography on early modern mortality statistics, particularly the work of Slack, Heitman, and McBride. This work aimed not to establish direct historical continuity between the Bills and modern epidemiology, but to illuminate how questions of data quality, transparency, and trust have long animated the ethics and politics of managing epidemic disease. The comparative references to COVID-19 are treated throughout as heuristic analogies rather than claims of methodological equivalence.

We argue that A Journal of the Plague Year can be read, in this analogical sense, as an early literary engagement with evidence-based reasoning at the intersection of literature, medicine, and public health. In an era marked by renewed attention to dashboards, case counts, and excess mortality during the COVID-19 pandemic, revisiting Defoe's engagement with the Bills of Mortality sharpens our understanding of recurrent tensions that remain central to epidemic governance [12].

The historical context of the Great Plague of London, the Bills of Mortality, and Defoe's project

The last major outbreak of bubonic plague in England, the Great Plague of London (1665), killed an estimated 100,000 people, roughly a quarter of the capital's population. The plague unfolded unevenly across the rapidly growing parishes of the city. It spread first in the suburban areas outside the old walls, such as St. Giles-in-the-Field, before moving inwards and reaching a devastating peak in the late summer and early autumn of 1665. What worried contemporary observers was not only the scale of mortality but also its spatial and temporal patterning, noticed week by week and parish by parish. They watched the progress of the disease as it appeared in specific neighborhoods, a possibility made available by a distinctive institutional innovation - the London Bills of Mortality, which transformed scattered individual deaths into a regular printed record of the city's vital events.

The Worshipful Company of Parish Clerks compiled and published the Bills of Mortality as weekly broadsheets from the early 17th century onward [6,7]. These broadsheets listed burials in each London parish on one side and the total deaths attributed to specific causes, including the "Plague," on the other. Surviving Bills from 1665 record thousands of deaths per week, with individual weeks in September reporting 7,165 plague deaths across more than 120 parishes at the height of the epidemic. Modern historians regard such figures as seriously underestimating the true mortality, owing to systematic undercounting of religious minorities and the urban poor [8,13-15]. A contemporary commentator noted that these sheets, sold for a penny and posted in public places, functioned as "of very great use ... it giveth a general notice of the Plague, and a particular Accompt of the places which are therewith infected, to the end such places may be shunned and avoided" [14]. They offered early modern Londoners a crude but influential form of epidemic intelligence, allowing them to make informed decisions about their safety and movements during the outbreak.

Historians of medicine and public health have emphasized that the London Bills of Mortality represent an early precursor of systematic mortality surveillance, emerging at the intersection of ecclesiastical record-keeping, civic governance, and commercial print culture. Slack has shown that, across the Tudor and Stuart periods, English state and city authorities increasingly relied on numerical information such as plague counts, parish totals, and trends over time to justify measures such as quarantine, the shutting up of houses, and the establishment of pest-houses [8,16]. The Bills were nonetheless products of their institutional context. Causes of death were certified by "searchers" of the dead and recorded by parish clerks. Certain groups, including nonconformists, Jews, and unbaptized infants, were often underrepresented. Whole families could sometimes persuade officials to register plague deaths under less alarming diagnoses, since a recorded plague death meant that the house was shut up [8]. Recent work has underlined that, despite these limitations, the weekly Bills gave Londoners a kind of "ever-changing plague map of the city," allowing them to adjust their movements, avoid certain parishes, or decide whether to remain in or flee from the capital [17,18].

This is the background against which A Journal of the Plague Year by Daniel Defoe must be understood. Published in 1722, more than 50 years after the 1665 outbreak, the work is narrated by "H.F.," a saddler living in London during the visitation of plague, and presents itself as a first-person chronicle based on observations or memorials of the year. Defoe himself had not yet been born in 1665, but he carefully constructed his narrative from a mixture of printed Bills of Mortality, municipal orders, medical treatises, and oral accounts, together with his own later reflections on plague and public health [3,5]. Scholars have long debated the precise status of the journal, whether as historical novel, documentary fiction, or quasi-ethnographic record. There is broad agreement, however, that Defoe engaged deeply and systematically with the numerical and documentary sources available to him [2,3].

In the Journal, the Bills of Mortality are not merely background noise but structural devices that organize the narrative of the epidemic's advance. Recent analysis has shown that the early chapters are arranged around a sequence of weekly Bills, with H.F. commenting on the first appearance of plague deaths in outer parishes, the subsequent increases in both "plague" and "spotted fever," and the ominous crossing of the boundary into the city within the walls [16]. Defoe's narrator repeatedly cites specific figures such as weekly totals, parish counts, and comparative numbers between weeks, then juxtaposes them with his observations in the streets and markets, using the Bills both to confirm and to question what he actually sees [1].

A striking instance of H.F.'s interpretive work occurs in his discussion of the diagnostic ambiguity between "spotted fever" and plague. Rather than accepting the official category at face value, he combines figures from both columns when estimating the true number of plague deaths in a given week. He observes that "a fraud lay in the parish officers, searchers, and persons appointed to give account of the dead," alleging that they were sometimes bribed "to procure ... the dead persons to be returned as dying of other distempers" rather than of plague [1]. In this move, H.F. effectively performs an ad hoc correction for under-reporting, treating the published Bills as a baseline that must be interpreted, supplemented, and corrected, rather than as a finished record of the epidemic.

Defoe's project thus occupies a crucial position in the history of epidemic representation. On one hand, he inherits a mature system of weekly mortality reporting that, by 1665, had become closely tied to the governance of plague, and he uses its data to reconstruct the temporal and spatial dynamics of the outbreak. On the other hand, he exposes the epistemic and ethical tensions embedded in this system, including the gap between counted and uncounted deaths, the political uses of numbers, and the anxieties of readers who must decide how to act in the face of imperfect information [2,3]. In this analogical sense, A Journal of the Plague Year offers historians of medicine and public health not only a vivid literary account of the Great Plague but also a sustained reflection on the promises and pitfalls of early modern mortality surveillance, one that resonates with contemporary debates about data, trust, and decision-making in epidemic crises [18].

The Bills of Mortality as proto-surveillance

The London Bills of Mortality were, in formal terms, single-sheet weekly publications produced by the Worshipful Company of Parish Clerks. They listed, on one side, the number of deaths in each of roughly 130 parishes, and, on the other, the total deaths by cause, including "Plague." As historians of medicine have shown, the Bills represented a remarkable logistical achievement, with data collected early in the week, centrally collated, printed, and distributed on a fixed schedule [8,12,13]. During a typical week of the 1665 outbreak, readers could quickly see that 1,414 individuals had succumbed to the "plague," 61 to "consumption," and one individual had been found dead in the fields at St. Mary Islington. The epidemic thus became legible as a pattern of numbers rather than a series of isolated reports. Bell explicitly praised the Bills, characterizing them as of very great use, giving a general notice of the plague and a particular account of the infected places [14]. He suggested that such places should be shunned and avoided, underscoring their function as both an early warning system and a record.

Recent historical work has emphasized that the Bills were not merely lists of the dead but instrumental methods of what we might now, anachronistically but usefully, call epidemic surveillance. Heitman's reconstruction of their emergence shows that by the late 16th century, aldermen of London already required weekly reports of deaths and causes to monitor the city's health and act before plague outbreaks became visible to the public [15]. In a 1591 manuscript bill preserved at the Folger Shakespeare Library, every parish is listed in alphabetical order, with separate columns for "Burials" and "Plague," even in a week with no plague deaths. This indicates that the authorities were committed to tracking all deaths by cause over time. These internal reports were initially secret state documents, used by the city and crown to gauge the economic and military strength of London and to decide when to impose quarantines or other restrictions. Only later, in the early 17th century, were the Bills printed regularly for public circulation, turning what had been an elite intelligence document into a commercially sold public-health news service [15].

From the perspective of the history of epidemiology, the Bills of Mortality represent a significant step towards population-level surveillance, though the comparison should be understood as heuristic rather than strict. They aggregated individual deaths into weekly series, broken down by geography and cause. These data made it possible for people to see when plague deaths began to rise, which parishes were hit the hardest, and when the epidemic seemed to be slowing down [8]. Heitman stresses that the dual tracking of total burials and plague-specific deaths was crucial [15]. Parishes varied greatly in population and density, so absolute counts of plague deaths alone could mislead. Comparing plague deaths against all burials offered a rough sense of proportional impact. The speed and regularity of the system, since the data were collected weekly, collated to a fixed deadline, and printed to a standard format, made it possible for officials and citizens to follow the "curve" of the epidemic in something close to real time. Historians have drawn a heuristic parallel between this feature and the role of COVID-19 dashboards in the 21st century, while noting that modern surveillance also involves standardized case definitions, laboratory confirmation, and statistical modeling, all of which were entirely absent in 1665 [10-13].

At the same time, both contemporaries and modern historians have been strongly aware of the limitations and biases of this early system [16]. The data depended on the judgments of "searchers" and parish clerks, who could misdiagnose causes of death. Nonconformists, Jews, and unbaptized infants were often excluded from the Church of England burial system and, therefore, underrepresented. Families had incentives to conceal plague deaths to avoid quarantine or stigma, and they sometimes persuaded officials to record these deaths under less alarming labels such as "spotted fever" [8,16]. Bell has argued that although the Bills were created "expressly to forewarn of plague," their use soon extended to other questions, such as patterns of accidents, violence, and chronic disease, which illustrates both their flexibility and their vulnerability to misinterpretation [17]. Contemporary commentators, including Samuel Pepys, frequently expressed skepticism about their accuracy even while relying on the weekly figures to gauge the severity of the "sickness" and to decide whether to leave the city or remain [16,18].

A Journal of the Plague Year by Defoe engages with this apparatus of counting from its very first pages. H.F. uses the Bills of Mortality as the narrative backbone for the early stages of the 1665 outbreak. The first part of the journal is organized around a sequence of weekly Bills, with detailed commentary on the rising number of plague burials in suburban parishes such as St. Giles-in-the-Field, St. Andrew's, St. Bride's, St. James, and Clerkenwell. As the Death by Numbers project has argued, H.F. treats the Bills as a kind of "tabular map" that tracks the eastward advance of the plague towards the city within the walls. He identifies, for instance, the moment when the first person "died (of plague) within the walls, in the parish of St Mary Woolchurch," as a turning point for the anxiety of the people in the city [1,16].

A particularly telling passage shows how H.F. moves from reading the Bills to drawing his own epidemiological inferences. Reflecting on the 40-day quarantine imposed on infected houses, he writes: "Forty days is, one would think, too long for nature to struggle with such an enemy as this, and not conquer it or yield to it; but I could not think, by my own observation, that they can be infected so as to be contagious to others above 15 or 16 days at farthest; and on that score it was, that when a house was shut up in the city, where any one had died of the Plague, and nobody appeared to be ill in the family for 16 or 18 days after, they were not so strict, but that they would connive at their going privately abroad" (pp. 227-228) [1].

Here, H.F. does something more than describe. He compares a normative figure, the 40-day quarantine prescribed by the orders, with an empirical estimate, 15 or 16 days, that he has derived "by my own observation." He then connects that estimate to observed administrative practice, noting that local officials quietly allowed families out after 16 or 18 symptom-free days. The passage shows a narrator who reads the Bills, measures them against what he sees in the streets, and arrives at a revised working estimate that differs from official policy. We do not wish to overstate the novelty of this operation. Graunt and Petty were engaged in much more systematic quantitative work in the same years. Nevertheless, the passage is a clear literary instance of what we might, cautiously, call proto-epidemiological reasoning in the pages of an early 18th-century text about 1665.

Bias, under-reporting, and interpretation

The Bills of Mortality made the plague numerically visible, but they did so in ways that people at the time already suspected were incomplete. They often omitted deaths from certain groups, and they relied on the subjective assessments of parish officials. These features made it difficult to grasp the epidemic's true impact and contributed to confusion and delayed responses. Parish officials and "searchers of the dead" were responsible for determining causes of death and reporting them to the Parish Clerks' Company, but as mortality rose in 1665, their capacity was quickly overwhelmed. Contemporary observers and modern historians agree that it was virtually impossible to maintain accurate accounts when people "died by heaps and were buried by heaps," so that rising mortality overwhelmed any attempt at precise enumeration [8,12,19]. Modern reconstructions suggest that the Bills failed to record entire categories of deaths, since they did not include nonconformists, Jews, the impoverished, and those buried outside the traditional parish system. Even within the covered parishes, causes of death could be misdiagnosed or strategically reassigned, leading to significant inaccuracies in the recorded data and further complicating the understanding of mortality trends [8]. The result, as McBride's recent work stresses, was a dataset adequate enough to detect broad trends but far from a complete census of the city's mortality [16].

Under-reporting was not only an artifact of administrative overload and diagnostic uncertainty. It was also the result of social and economic incentives. Families had strong reasons to avoid having a death in their house labeled as "plague," since such a label might lead to the house being shut up, the neighbors shunning them, or the employer dismissing servants [8]. Defoe's narrator directly addresses this phenomenon when he alleges that "a fraud lay in the parish officers, searchers, and persons appointed to give account of the dead," who were sometimes bribed "to procure ... the dead persons to be returned as dying of other distempers" rather than the plague [1]. H.F. repeatedly treats deaths listed as "spotted fever" as probable plague cases and adds them to his estimates, effectively attempting to correct what he perceives as intentional under-reporting [16]. This narrative "recalibration" not only testifies to Defoe's awareness of bias in the Bills but also illustrates how a literate reader of the period could reinterpret official categories in light of experience and suspicion.

The Bills were also shaped by inequalities of information and power. Recent research from the Death by Numbers team and the University of Portsmouth shows that literate and wealthy Londoners such as Samuel Pepys could read and interpret the weekly numbers, adjust their movements accordingly, and even leave the city when they feared a spread of plague. Poorer residents, servants, and laborers who lacked both the literacy to interpret the Bills and the economic means to act on them would remain in the city, facing a significant risk of death. As McBride notes, the Bills "almost fostered a sense of self-surveillance and self-governance among those with the resources to respond, but for many others, ongoing exposure was unavoidable" [16]. Deaths among the poor were also more likely to be misclassified and under-reported, since enormous numbers of poor people were buried in mass graves. The worst-affected communities, therefore, skewed the apparent geography of the epidemic, distorting the understanding of who suffered most [8,16]. The apparent objectivity of the printed numbers masked social disparities in whose deaths were counted, how they were described, and who could put the information to use.

Defoe's Journal repeatedly stages the tension between official figures and the field experience of everyday life. As Connor remarks, H.F. states repeatedly that "the official data does not accord with his observations as he walks the city's streets and that they seriously underestimate the deaths due to plague" [18]. Defoe offers the following two explanations through H.F.: first, that accurate enumeration was impossible owing to the sheer scale and speed of death; and second, that deliberate concealment and mislabelling suppressed the true plague toll [1,18]. A revealing passage shows how H.F. interpreted this gap: "It is easy to see that the shutting up of houses was no way to be depended upon as a sufficient method for putting a stop to the infection; because, as I have said elsewhere, many of those that stole out of those infected houses had the plague really upon them, though they might really think themselves sound; and some of these were the people that walked the streets till they fell down dead, not that they were suddenly struck with the distemper as with a bullet that killed with a stroke, but that they really had the infection in their blood long before" (pp. 192-193) [1].

The passage is significant in two ways. First, it shows H.F. reasoning about transmission in terms that, to a modern reader, sound like asymptomatic or presymptomatic infection, even though Defoe did not have that vocabulary and his theoretical frame remained miasmatic. Second, it connects the inadequacy of the Bills to the inadequacy of the policy response. If people are already infectious "long before" any visible sign, then both household quarantine and parish-level death counts are systematically lagging the real state of the epidemic. We do not suggest that Defoe understood the pathophysiology of plague. Modern science attributes transmission chiefly to rats and fleas in the case of bubonic plague, and to respiratory droplets in the case of the pneumonic form. What the passage does show is a narrator who has noticed, by observation rather than by theory, that the official evidence base was structurally delayed relative to the disease it was supposed to monitor.

H.F. takes a related position on the printed figures themselves. He "took it for granted upon the whole" that the true mortality was substantially higher than what appeared in the Bills during weeks when "it was certain" many more people died from the plague than the sheets recorded [1,16]. Rather than rejecting the Bills outright, he treats them as a baseline that must be interpreted, supplemented, and corrected. This stance aligns with Rosenberg's broader argument that epidemic numbers are always embedded in social negotiations about what counts, who counts, and how uncertainty is handled [10].

The situation Defoe describes, seen from the vantage point of contemporary epidemiology and health policy, is recognizable without being identical. Modern analysts of COVID-19 or other epidemics routinely distinguish between "reported" and "excess" deaths, debate the impact of diagnostic criteria and testing capacity on case counts, and worry about the political uses of optimistic or pessimistic numbers. As has often been observed, in an epidemic "the numbers become the narrative," shaping how events are perceived and remembered [10,12,13]. Lau makes this point explicit for COVID-19 when he observes that, in a plague year, "the numbers are the narrative," comparing how Londoners followed the Bills in 1665 to how 20th-century citizens have watched daily dashboards of coronavirus deaths [11]. H.F. anticipates this logic, in the analogical sense we have been developing, by treating the Bills as necessary but insufficient evidence. The Bills are indispensable for tracking the epidemic, yet they must be read with awareness of bias, omission, and manipulation. In this sense, A Journal of the Plague Year offers not only an early modern case study of under-reporting and uncertainty, but also a reflection on the epistemological status of epidemic data that remains pertinent to current debates about surveillance and trust.

Risk perception and public health action

The Bills of Mortality did not simply describe the Great Plague. They actively shaped how individuals and authorities perceived risk and decided how to act. The Death by Numbers project has emphasized that Londoners "rapidly embraced the Bills as a tool for evaluating their risk of imminent death," using weekly death figures to judge whether the "sickness" was approaching their parish or receding from it. The Bills translated scattered reports of illness into a single comparative frame, so that readers could observe when plague deaths in specific outer parishes doubled from one week to the next, or when burials within the city walls remained relatively low, and then adjust their behavior accordingly [16]. Mortality numbers became, in this sense, a form of public health information, although imperfect and unevenly understood. They gave people an opportunity to manage their exposure to danger, within limits set by factors the Bills did not capture, such as social interactions and living conditions.

Recent research on Samuel Pepys's use of the Bills provides a microhistorical illustration of this process. According to McBride, Pepys's diary shows that he carefully recorded weekly plague figures and compared them across weeks and parishes to decide when to send his wife and household out of London, when to alter his routes to avoid badly affected areas, and when to resume social visits as numbers began to fall [16]. Pepys's behavior demonstrates how elite, literate Londoners could transform printed statistics into concrete choices, drafting wills at peak mortality, limiting contact when deaths surged, and returning to everyday life when the Bills suggested that the worst wave of plague had passed [16]. Municipal and royal authorities relied on the same mortality data to apply and justify interventions such as shutting up houses, restricting travel, banning gatherings, and directing the sick to "pest-houses" outside the city walls [8]. The counting of deaths in mortality Bills thus underpinned both personal strategies of self-protection and collective policies of containment.

A Journal of the Plague Year by Defoe offers a complementary narrative perspective on this dual role of numbers. As Connor argues, the journal can be read as "a study of risk management in a time of crisis," where the observations of H.F. are constantly juxtaposed with the official Bills [18]. H.F. describes the reactions of different groups to these numbers, from merchants who flee when weekly totals cross certain thresholds, to artisans who decide to stay because their work is tied to the local economy, to families who wait anxiously for a second or third bill to confirm whether an apparent decline in deaths is real or a temporary fluctuation [1,18]. The narrator himself oscillates between trusting the Bills as a guide to prudent action and distrusting them because of under-reporting and misclassification [1,16]. Connor interprets this attitude as Defoe's attempt to stage a tension between the knowledge grounded in personal observation and the emergent quantitative reasoning associated with John Graunt and William Petty [18]. The framing is heuristic rather than strictly historical, since Graunt and Petty's more systematic statistical work lies outside the journal's own horizon. It nonetheless captures how H.F. positions himself between direct experience and printed numbers.

A passage from the opening of the journal shows how the Bills translate into a personal dilemma. Reflecting on the first signs of the epidemic, H.F. records the advice he received from his brother and his own hesitations about whether to flee, which he weighs in the light of what he reads in the Bills. Later, describing the height of the outbreak, he notes the practical limits of the policy response as follows: "There was likewise violence used with the watchmen, as was reported in abundance of places; the people broke out, whether by force or by stratagem, even almost as often as they pleas'd ... those that did thus break out, were generally people infected, who in their desperation, running about from one place to another, valued not who they injur'd. People sicken'd so fast, and died so soon, that it was impossible and indeed to no purpose to go about to enquire who was sick and who was well, or to shut them up with such exactness as the thing required" (p. 84) [1].

The passage is doing several things at once. H.F. is reporting observed behavior (people breaking out of shut-up houses), attributing a causal pattern (desperation plus infection produces further transmission), and drawing a practical conclusion (the policy cannot be implemented with the precision its logic would demand). In the analogical vocabulary we have adopted, this is risk communication in reverse. The narrator uses concrete cases to argue that the authorities' own framework, which rests on identifying and isolating the sick, cannot be made to work under the epidemic conditions that the Bills themselves were tracking. H.F. is not proposing that quarantine be abandoned. He is arguing that it cannot deliver what it promises, and that part of the evidence for this conclusion is the gap between the policy's assumptions and the realities the Bills partially record.

In terms of public health action more broadly, the Bills served as a political and informational tool. A rise in plague figures in specific parishes could lead the magistrates to legitimate coercive measures such as shutting up houses with a painted red cross, enforcing quarantine, and prohibiting the movement of groups of people from infected to "clean" areas [8]. H.F. comments repeatedly on the use of mortality numbers to support these policies, even while he criticizes their implementation and effectiveness, arguing, for example, that shutting up houses often drove desperate residents to break out and spread infection more widely [1,18]. Rosenberg has argued that epidemics unfold as "dramas" in which numbers play a vital role, helping societies move from denial to recognition to action [10]. In 1665, the Bills provided the quantitative script. Only when deaths rose to certain levels could the authorities no longer ignore the presence of epidemic disease, and only then were the costly and disruptive interventions politically defensible. The rising numbers forced a shift in public perception and policy response to the crisis.

The capacity to translate numbers into action was, at the same time, unevenly distributed. McBride's study underscores that, although the Bills were public, many Londoners lacked the literacy, financial means, or social freedom to act on the information they contained [16]. Only wealthy householders could send their families to the countryside, adjust their travel, or temporarily close their businesses. Servants, casual laborers, and the urban poor often remained in crowded housing and continued working despite escalating death counts [16]. The journal foregrounds this asymmetry. H.F. describes poor families who die behind barred doors, unable to obtain food or medical help, alongside wealthier citizens who used both personal observation and the Bills to time their escape from the city [1,5]. Mortality numbers thus both enabled and exposed inequalities in risk management. The Bills informed decisions, but only some people had the capacity to make those decisions, often because of socioeconomic factors that limited their access to information and resources.

The interaction between the Bills, risk perception, and action in 1665 anticipates, in this analogical sense, patterns that recurred during the COVID-19 pandemic. Daily dashboards of deaths and cases structured how individuals and governments perceived danger, justified restrictions, and debated when to reduce controls [11]. Defoe's narrator depicts the struggle of Londoners and officials to interpret imperfect data, weigh personal observation against official figures, and navigate the ethics of coercive measures. The comparison is instructive, but not seamless. The instruments, the science, and the institutional frameworks differ. What recurs is the difficulty of translating imperfect numbers into action under pressure [10,18].

Medical and moral frameworks around the numbers

The numerical logic of the Bills of Mortality operated within a wider set of medical and moral frameworks that shaped how plague deaths were counted and interpreted. Explanations of plague during the early modern English period drew on a fusion of Galenic medicine, miasma theory, and providentialist theology. Corrupted air, disordered humors, and sinful behavior were all regarded as interlocking causes of epidemic disease [10,20,21]. Pannell's study of miasma in early modern England shows how smell, air, and "corrupt vapors" were thought to transmit disease, giving rise to practices such as fumigation, burning of infected goods, and leaving "ill-aired" places [20]. At the same time, sermons and devotional writings interpreted plague as a divine scourge sent to punish communal sin, particularly pride, greed, and lack of charity [22,23]. These medical and moral beliefs did not simply coexist. They informed each other, so that the management of air and regimen could be framed simultaneously as prudence and as an act of repentance.

The substantial body of English plague treatises of the period illustrates this hybrid "religio-medical" discourse. Healy and Cipolla have shown how Elizabethan and Stuart plague treatises combine practical advice about clean air, diet, fumigation, and quarantine with prescriptions for fasting, prayer, and moral reform [23-25]. Ratia's analysis of Stuart-period plague treatises demonstrates that many works fall into a "religio-medical" category, in which medical discussion of causes, contagion, and remedies is combined with biblical quotations, prayers, and exhortations to penitence [12]. One could find in the same tract advice on the regulation of sleep, avoidance of "corrupt airs," and the recommendation of penitence as "the best medicine against the pestilence," linking bodily regimen to spiritual discipline [8,12]. Slack underscores that the shift of some treatises towards more naturalistic explanations in 1665 did not eliminate providential thinking, but allowed the two registers to coexist, reflecting a spectrum of views about how divine and natural causes could interact [8,12].

In London during the Great Plague of 1665, moral interpretations of plague were closely tied to social order and to ideas of collective responsibility. Civic and ecclesiastical authorities regularly described plague as God's punishment for the sins of the city, especially those of the "disorderly" poor found in the suburbs beyond the walls [21]. Miller notes that a University of London research project on "discourses of the plague in early modern London" has shown how pamphlets and sermons portrayed pestilence as a "penal matter" directed at a sinful populace, and associated the disease with the "dirty, unruly poor" and with moral vices such as greed and lack of charity [22]. Thomas Dekker's plague satires repeatedly insist that "sin and not contagion causes the plague," arguing that only charity and repentance can settle divine wrath [22]. These moralizing discourses also helped to legitimate strict public health measures, particularly when particular groups were stigmatized as morally and physically "plaguey," making it easier to justify harsh quarantine, forced removal, or punitive restrictions on movement [21,22]. Medical ideas about contagion and miasma, refracted through a moral lens, were thus linked to specific groups and neighborhoods in ways that were not neutral.

This is the dense interpretive context within which H.F. reads and interprets the Bills. Numerical evidence in the journal is rarely allowed to stand alone. H.F. repeatedly reflects on questions of providence, duty, and trust in God when he has to decide whether to stay in or flee from London, interpreting his hesitations and "accidents" as possible signs of divine will [1]. He cites Psalm 91, "there shall no evil befall thee, neither shall any plague come nigh thy dwelling," and reads the verse as a promise of protection for those who make God their refuge, using it to justify his decision to remain in the city [1,26]. In the same pages, he engages seriously with medical ideas of contagion and miasma, discussing whether infection is carried "by the air only, by carrying with it vast numbers of insects and invisible creatures," and recounting contemporary debates about Athanasius Kircher's theory of "worms" and "seminal seeds" of disease [1,27].

This layered interpretive stance has direct implications for how H.F. handles the Bills. When plague deaths in a given week rise sharply, H.F. is willing to read the figures not only as an indicator of contagion but also as a sign of divine displeasure. When they fall after days of public fasting and thanksgiving, he is willing to entertain a providential reading. He also notes, without resolving the tension, that the same rise or fall can be read in miasmatic, humoral, or purely empirical terms. The Bills are therefore embedded in his narrative as a shared object of multiple interpretations, not as a neutral dataset to be plugged into a single framework. Huang's recent literary-historical work makes a complementary point, arguing that the journal uses sensory descriptions of air, smell, and sound to mediate between material and spiritual understandings of plague, so that foul air and fearful cries become signals of both physical danger and moral crisis [28]. A related study of quarantine debates highlights how Defoe's contemporaries argued about whether strict quarantine reflected a lack of trust in God's providence or an appropriate application of God-given reason [17]. H.F. registers these debates explicitly. He criticizes the shutting up of houses as ineffective and cruel, yet he frames the suffering of the "shut-up" poor as a test of Christian charity and civic responsibility [1,18].

The practical point of our argument is that, in 1665 and in the journal, mortality counts were never presented to readers as raw numbers. They arrived already interpreted through theological, humoral, and experiential lenses, and the Bills were read accordingly. A sudden rise in plague deaths could be confirmation of the city's sinfulness, a change in the air, or evidence that more parishes had been reached. A sustained decline after days of public prayer could be read as evidence of divine favor, as miasmatic clearing, or as an epidemic running its natural course [12,22]. H.F. follows the weekly figures closely, but he also takes note of days of public fasting and thanksgiving and invites readers to observe correlations between religious observances and changes in the Bills [1,5]. In Rosenberg's terms, the "dramaturgy" of epidemics always involves this dual coding of data, so that numbers serve at once as technical indicators and as moral symbols through which communities make sense of suffering [10]. This dual coding matters for our central argument. The interpretive operations performed by H.F. on the Bills, whether correction for under-reporting, inference about transmission, or criticism of policy, were carried out within a thick medical and moral framework that shaped both what he saw in the numbers and what he took them to mean.

Parallels with COVID-19

The issues Defoe explores through H.F., such as how to interpret imperfect mortality data, how to balance personal risk against public duty, and how far to trust official guidance, re-emerged with particular force during the COVID-19 pandemic. Seventeenth-century Londoners followed the weekly Bills of Mortality to decide whether to stay, flee, or alter their movements. Twenty-first-century citizens monitored daily dashboards of cases, deaths, and reproduction numbers to judge when it was safe to visit relatives, travel, return to workplaces, or send children to school [10,11,16]. Quarantines and the shutting up of houses in 1665 had their functional counterparts in the lockdowns and travel bans of 2020 and 2021. In both periods, the data collected remained open to contestation and distrust.

These similarities are heuristic rather than strict. The instruments, the science, and the institutional frameworks differ substantially. Modern epidemiology rests on standardized case definitions, laboratory confirmation, statistical modeling, and formal structures of ethical oversight that did not exist in 1665. The Bills of Mortality were compiled by parish clerks on the basis of reports from "searchers" of the dead, not by trained epidemiologists working from confirmed laboratory diagnoses. What recurs across the two periods is therefore not a continuity of method, but a persistence of problems, including the gap between counted and uncounted deaths, the unequal capacity to act on imperfect information, and the role of numbers in legitimating coercive measures.

The COVID-19 experience makes this persistence visible. Defoe's concerns about misclassified plague deaths, overwhelmed parish officials, and the invisibility of the poorest Londoners in the Bills of Mortality have analogs in contemporary debates about limited testing, asymptomatic infections, and cross-national differences in how COVID-19 deaths were counted [16,18]. In both cases, mortality figures were unevenly translated into protective decisions. Those with resources could more easily withdraw from exposure, while essential workers and marginalized communities remained disproportionately at risk [10,16]. The structural asymmetry that McBride identifies in Pepys's use of the Bills, where literacy and economic means determined who could act on the information, is recognizable in 21st-century patterns of differential exposure during lockdown [16].

Read in this way, A Journal of the Plague Year offers a historical reference point rather than a direct precedent for current debates on surveillance, trust, and transparency. It does not show that modern problems were already "solved" or even fully understood in 1665. Instead, it shows that the practice of counting the dead has long been entangled with questions of power, belief, and the ethics of public health. Reading Defoe alongside contemporary pandemic debates can therefore sharpen, rather than replace, the critical questions we bring to modern epidemic data.

Conclusions

A Journal of the Plague Year is not an epidemiological treatise, nor a history of the Great Plague in any strict sense. It is a literary work that thinks with numbers. Read in this way, it offers historians of medicine and public health something that more conventional sources rarely provide as follows: a sustained, first-person attempt to reason through imperfect mortality data in real time, placing printed figures in dialogue with street-level observation, policy debate, and competing medical and moral frameworks. The narrator, H.F., does not treat the Bills of Mortality as a definitive truth. He compares them with what he sees, suspects under-reporting, infers missing cases, corrects official categories, and draws practical conclusions about transmission and containment. His reasoning is not modern epidemiology. It proceeds without standardized definitions, without laboratory confirmation, and within a dense web of humoral and providential assumptions. What it shares with modern epidemiology, analogically rather than historically, is the recognition that epidemic numbers are always contested, always partial, and always interpreted through social, political, and ethical commitments.

This recognition is what makes the journal still worth reading in the aftermath of a global pandemic. The questions that preoccupied H.F., about whose deaths get counted, how numbers are produced and circulated, who can act on them, and what moral weight they carry, are the same questions that animate contemporary debates about surveillance, transparency, and trust. Revisiting Defoe does not resolve those questions. It reminds us that they are older than the instruments we currently use to answer them, and that the literary handling of epidemic evidence has something distinctive to contribute to an understanding of the present.
